# P-1636. Real World Outpatient Utilization of Novel Anti-MRSA Antimicrobials: Patterns in Prescribing and Cost of Oral and Long-Acting Injectable Anti-MRSA Therapies in United States Medicaid Beneficiaries

**DOI:** 10.1093/ofid/ofae631.1802

**Published:** 2025-01-29

**Authors:** Jessica C O’Neil, Ebbing Lautenbach

**Affiliations:** University of Pennsylvania, Philadelphia, Pennsylvania; University of Pennsylvania, Philadelphia, Pennsylvania

## Abstract

**Background:**

Methicillin Resistant Staph Aureus (MRSA) isolates with resistance to traditional oral anti-MRSA antimicrobials represent a major public health concern. Since 2014, the United States (US) Food and Drug Administration (FDA) approved three oral (tedizolid [2014], delafloxacin [2017], omadacycline [2018]) and two long-acting injectable (LAI) antimicrobials (dalbavancin [2014], oritavancin [2014]) with potent activity against MRSA. Little has been reported about the real-world outpatient utilization and cost of these important therapies.

**Figure:**
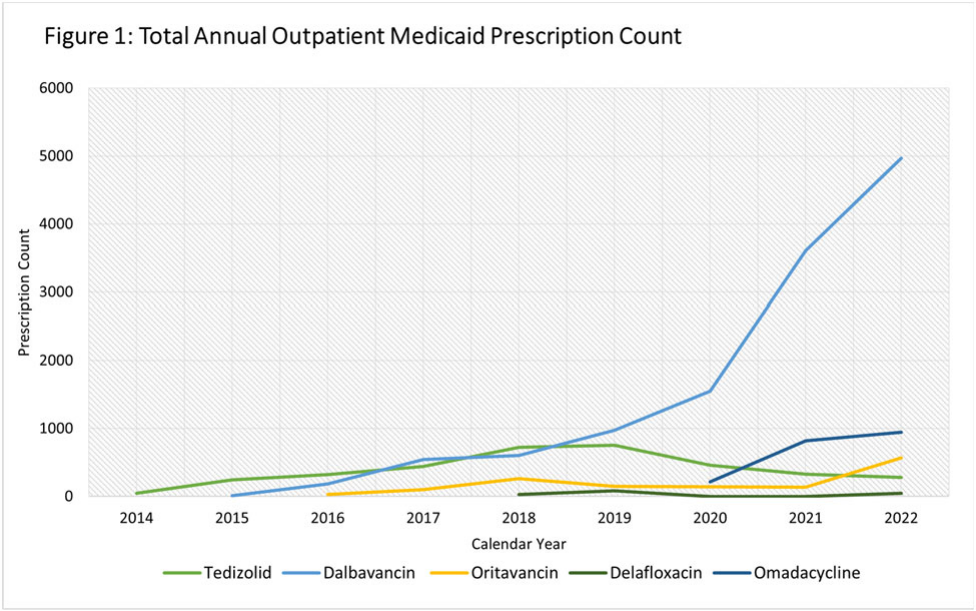

**Methods:**

We conducted a retrospective analysis of utilization and cost of these 5 antimicrobials between January 1^st^, 2014 and December 31^st^, 2022 in the Medicaid State Drug Utilization Database (SDUD). The SDUD contains all covered outpatient medications excluding those reimbursed under bundled services and suppresses medication data for years with fewer than 11 prescriptions. Annual cost was calculated as the sum of Medicaid and non-Medicaid reimbursement. We approximated the annual per prescription cost (APPC) as the quotient of total cost and prescription count.

**Figure:**
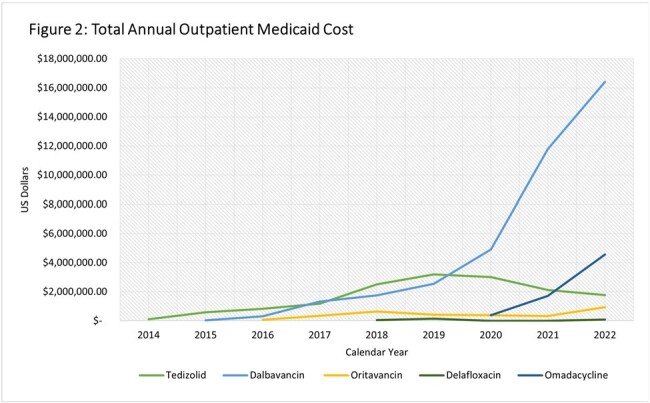

**Results:**

Trends in utilization, total cost and APPC are depicted in Figures 1, 2 and 3 respectively. There was a 1–2-year lag between FDA approval and utilization. Dalbavancin utilization and cost rose from 13 prescriptions and $26,224 in 2014 to 4970 prescriptions and $16,403,304 in 2022, while oritavancin increased from 27 to 566 prescriptions and from $62,309 in $944,657costs. The dalbavancin APPC increased over the study period ending in 2022 at $3,300 while the oritavancin APPC declined to $1669 in 2022. Of the oral antimicrobials, tedizolid utilization and total cost peaked in 2019 at 755 prescriptions and $3,193,571, after which total cost and utilization fell to 283 prescriptions and $1,773,238 by 2022. Delafloxacin utilization and total cost remained low while omadacycline had an increasing trend in utilization, total cost and APPC in the three years it was included.

**Figure:**
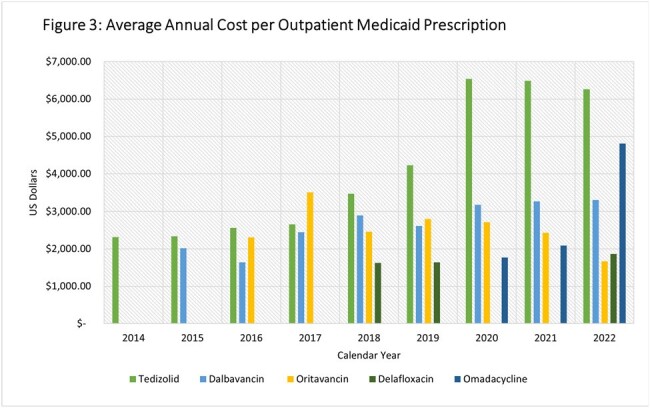

**Conclusion:**

Utilization and total cost of dalbavancin rose substantially over the study dwarfing that of oritavancin and the novel oral antimicrobials by the latter half of the study period. Omadacycline, the newest approved antimicrobial included, demonstrated a prompt rise in utilization and total cost.

**Disclosures:**

**All Authors**: No reported disclosures

